# Identification of Crucial Genes and Pathways in Human Arrhythmogenic Right Ventricular Cardiomyopathy by Coexpression Analysis

**DOI:** 10.3389/fphys.2018.01778

**Published:** 2018-12-06

**Authors:** Peipei Chen, Bo Long, Yi Xu, Wei Wu, Shuyang Zhang

**Affiliations:** ^1^Department of Cardiology, Peking Union Medical College Hospital, Chinese Academy of Medical Sciences and Peking Union Medical College, Beijing, China; ^2^Central Research Laboratory, Peking Union Medical College Hospital, Chinese Academy of Medical Sciences and Peking Union Medical College, Beijing, China

**Keywords:** arrhythmogenic right ventricular cardiomyopathy, weighted gene coexpression network analysis, biomarker, differentially expressed genes, gene set net correlations analysis

## Abstract

As one common disease causing young people to die suddenly due to cardiac arrest, arrhythmogenic right ventricular cardiomyopathy (ARVC) is a disorder of heart muscle whose progression covers one complicated gene interaction network that influence the diagnosis and prognosis of it. In our research, differentially expressed genes (DEGs) were screened, and we established a weighted gene coexpression network analysis (WGCNA) and gene set net correlations analysis (GSNCA) for identifying crucial genes as well as pathways related to ARVC pathogenic mechanism (*n* = 12). In the research, the results demonstrated that there were 619 DEGs in total between non-failing donor myocardial samples and ARVC tissues (FDR < 0.05). WGCNA analysis identified the two gene modules (brown and turquoise) as being most significantly associated with ARVC state. Then the ARVC-related four key biological pathways (cytokine–cytokine receptor interaction, chemokine signaling pathway, neuroactive ligand receptor interaction, and JAK-STAT signaling pathway) and four hub genes (*CXCL2, TNFRSF11B, LIFR*, and *C5AR1*) in ARVC samples were further identified by GSNCA method. Finally, we used *t*-test and receiver operating characteristic (ROC) curves for validating hub genes, results showed significant differences in *t*-test and their AUC areas all greater than 0.8. Together, these results revealed that the new four hub genes as well as key pathways that might be involved into ARVC diagnosis. Even though further experimental validation is required for the implication by association, our findings demonstrate that the computational methods based on systems biology might complement the traditional gene-wide approaches, as such, might offer a new insight in therapeutic intervention within rare diseases of people like ARVC.

## Introduction

As one inherited cardiomyopathy involved into the right ventricle primarily, arrhythmogenic right ventricular cardiomyopathy (ARVC) is characterized by cardiomyocytes’ fibrofatty replacement (Calkins et al., 2017; Corrado et al., 2017). The prevalence of the general population is estimated to be 1:5000 to 1:1000 (Rampazzo et al., 1994; Peters et al., 2004). ARVC is a common cause resulting in young people’s sudden cardiac death (SCD) (Bagnall et al., 2016; Finocchiaro et al., 2016), and SCD may be the first manifestation of the disease (Dalal et al., 2005; Gupta et al., 2017). The detailed clinical features of ARVC were first described in 1982 (Marcus et al., 1982), followed by reports showing that pathogenic genetic mutations occurred in more than 60% of patients (Bhonsale et al., 2015), and high genetic heterogeneity and dominant inheritance most commonly causes the genetic etiology to patients with ARVC. Currently, the most effective treatment of ARVC is implantable cardioverter defibrillator (ICD), and early diagnosis and detection is needed. Hundred of genes were estimated to be involved into ARVC molecular mechanism, leading to a more complex ARVC diagnosis and prognosis. However, molecular genetic diagnostic yield is known to be highly variable, ARVC-related targeted genes is still unclear (Bhonsale et al., 2015). For obtaining a better understanding of complicated mechanism and exploring potential ARVC eigengenes, we used the weighted gene coexpression network analysis (WGCNA) approach for the research analysis.

At present, gene expression profiles have been utilized for identifying genes related to diagnosis or progression in the cardiovascular field (Boileau et al., 2018; Molina et al., 2018; Theriault et al., 2018). However, while genes that have similar patterns of expression might be related in a functional way, most studies are only interested in screening for differentially expressed genes (DEGs) and ignoring highly interconnected genes (Tavazoie et al., 1999). WGCNA is one system biology approach used to describe pattern of correlation between genes in RNA sequencing data or microarray. It is one algorithm to discover highly related gene clusters (modules) and identifying phenotypically related modules or gene clusters (Langfelder and Horvath, 2008). At present, many studies in the cardiovascular field have revealed genes related to the phenotype and differentiation stages by the method of WGCNA (Liu et al., 2017; Wang T. et al., 2017; Tang Y. et al., 2018). For instance, *ZEB1* was found to be essential for early cardiomyocyte differentiation (?). *FKBP11* could act as one critical regulator within acute aortic dissection (Wang T. et al., 2017). In the research, we try to screen the DEGs, and establish the coexpression network to find the key biological pathways and hub genes that are involved within ARVC state.

## Materials and Approaches

### Data Gathering

We downloaded the gene expression profile in the database, Gene Expression Omnibus (GEO)^1^. The dataset GSE29819 from an Affymetrix Human Genome U133 Plus 2.0 Array [transcript (gene) version] (Affymetrix, Santa Clara, CA, United States) was utilized in this study. This dataset contains 6 ARVC specimens derived from heart transplantation candidates were compared with six non-failing donor hearts (NF) which could not be transplanted due to technical reason. From the hearts, both right ventricle (RV) and left ventricle (LV) myocardial samples were analyzed using Affymetrix HG-U133 Plus 2.0 arrays.

### Research Design and Data Preprocessing

**FIGURE 1:**
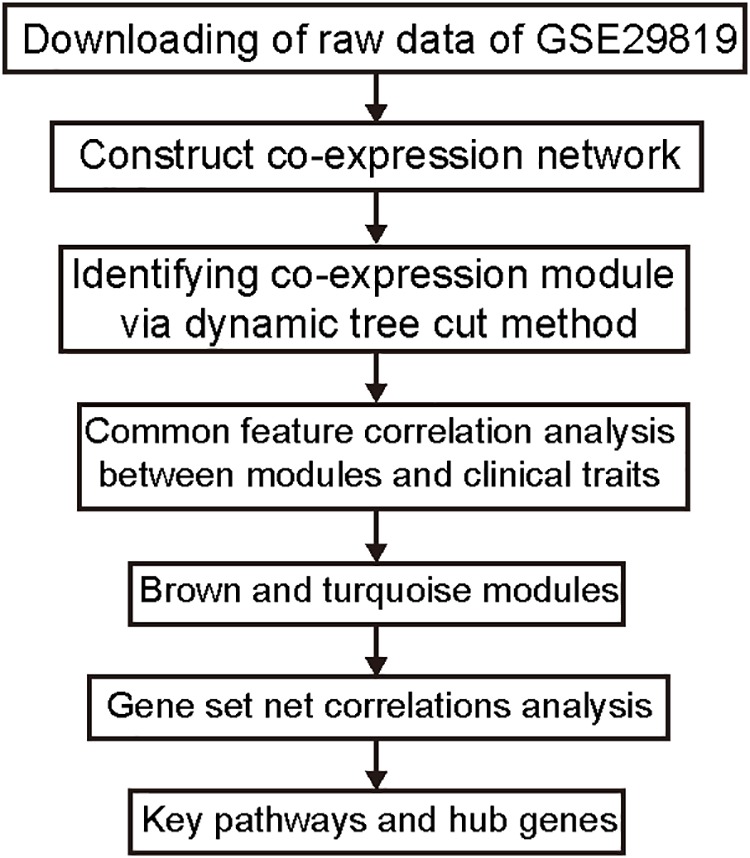
Study flow diagram. Data preparation, processing, and analysis are shown in the flow diagram.

**FIGURE 2:**
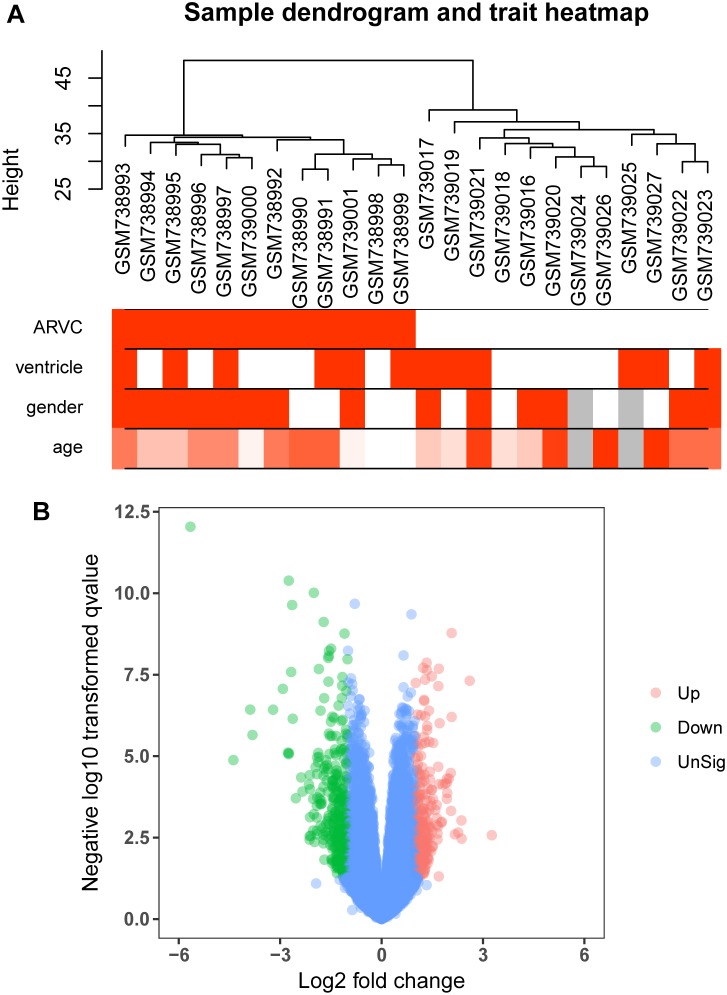
Clustering dendrogram and the clinical traits, as well as identification of differentially expressed genes (DEGs) in ARVC tissues. **(A)** Clustering dendrogram and clinical traits of GSE29819. The clustering was on the basis of the expression data of DEGs between ARVC samples and non-failing donor myocardial samples. The red color represents the right ventricle and men. The color intensity was in proportion to the older age. **(B)** The volcano plot of all DEGs.

One flow diagram (Figure 1) shows the research design. We calculated the raw expression data using the preprocessing steps as follows: robust multichip average background correction, log2 transformation, quantile normalization as well as median polish algorithm summarization utilizing “affy” R package (Gautier et al., 2004). The Affymetrix annotation files annotated the probes. Sample clustering was used to assess the quality of microarray in accordance with distance between various samples within Pearson’s correlation matrices. All samples from the data set were not removed in the subsequent analysis (Figure 2A).

### DEGs Screening and Principal Component Analysis

**FIGURE 3:**
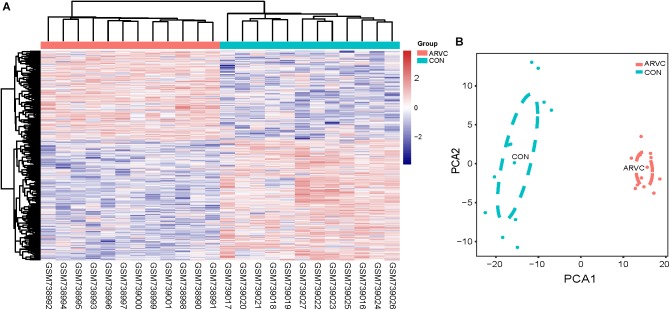
**(A)** Heatmap showing significantly differentially expressed protein-coding genes of ARVC and non-failing donor myocardial tissues. In the heatmap, samples are sorted by columns, and genes by rows. Cyan square represented Control group, and red square represented ARVC group. **(B)** PCA scores trajectory plots showing obvious differences resulting from the ARVC and Control groups. Cyan point, control group; red point, ARVC group.

We use the “limma” R package (Ritchie et al., 2015) for screening DEGs between ARVC samples and non-failing donor myocardial samples. The false discovery rate (FDR) < 0.05 and |log_2_fold change (log_2_^FC^)|>1 were selected to be cutoff criteria (Figure 2B). A heatmap was plotted using the pheatmap package (Figure 3A). Two features were extracted from the genes of each group using an unsupervised principal component analysis (PCA) method (Figure 3B).

### Construction of Coexpression Network

**FIGURE 4:**
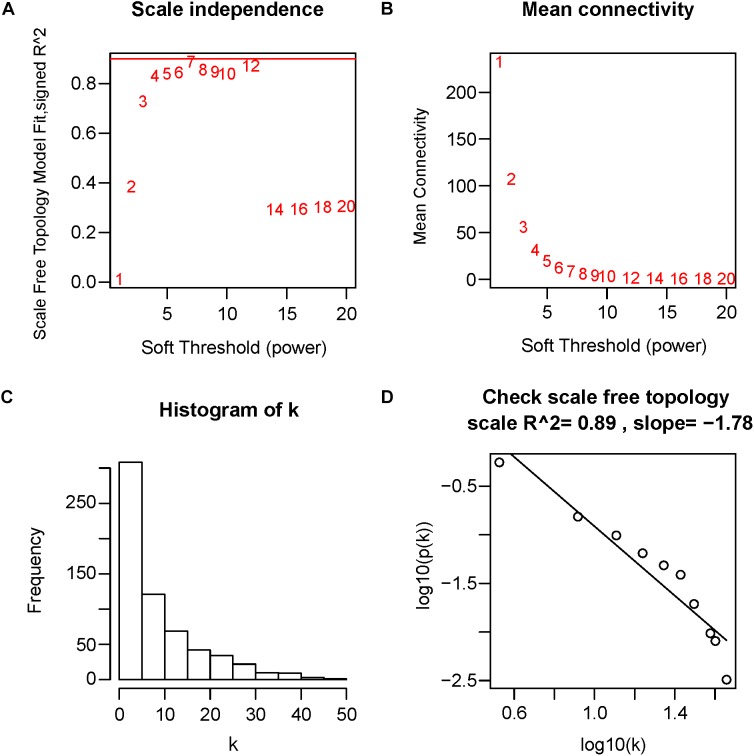
Determination of soft-threshold power in WGCNA. The scale-free topology index and the mean connectivity for each power value between 1 and 20 are shown in **(A,B)** panels, respectively. The histogram of connectivity distribution and the scale-free topology are shown in **(C,D)** panels, respectively.

First of all, DEGs expression data profile was tested for confirming that they meet the subsequent analysis requirements. Secondly, package of “WGCNA” within R was used for constructing DEGs coexpression network (Horvath and Dong, 2008; Mason et al., 2009). WGCNA is one statistical method which sets up gene sets (modules) from gene expression data that are observed using unsupervised clustering, briefly, it assigns a connection weight between pairs of genes in network on the basis of one scale-free topology (SFT) criterion as well as tries to recognize related modules using one soft threshold to correlations between pairs of genes in one network, and thus does not rely on *a priori* defined gene sets. Within one biological network that has an SFT, gene relationship distribution adheres to the law of power decay, for example, the genes that have the maximum number of connections appears at the minimum frequency (van Noort et al., 2004; Barabasi, 2009). Function firstly calculates one pairwise correlation matrix for all gene sets, then, it computes a nearby matrix by elevating matrix to one soft threshold power (β). Pearson’s correlation matrix was conducted for each pairwise gene and used the power function a_mn_ = |c_mn_|^β^ (c_mn_ = Pearson’s correlation between gene n and gene m; a_mn_ = adjacency between gene n and gene m) was used. The β utilized for transforming similarity matrix is chosen when resultant network approximates one scale-free topology the best. The transformation process of correlation matrix to approximate architecture of scale-free network starts by elevating matrix to one range of β (such as β = 1–20 in Figure 4A) for producing one series of adjacency matrices. The connectivity of genes could be defined as significance of one specific gene in one network of coexpression and is computed only by summing all rows within one adjacency matrix. For selecting the most suitable β value, linear model fit (R^2^) between log(k) and log(p(k)) is computed from all adjacency matrices, where k = connectivity, p(k) proportion of genes with connectivity k (Figures 4A,D). Perfect agreement with SFT could generated an *R*^2^ = 1, nevertheless, if the SFT fit index for reference data set reached above-0.8 values for the lower power (<30) (Langfelder and Horvath, 2017), as power of threshold defined in. For example, within WGCNA signed, β values which generated one *R*^2^ > 0.8 is regarded to be one fit acceptable for SFT, and usually chose the one which first approached the highest value. Fitting to a power-law has been a frequent strategy of WGCNA in previous researches (Sikri et al., 2018; Tang J. et al., 2018; Yuan et al., 2018; Zhou et al., 2018), and is also the strategy followed in this study. The value of R^2^ was 0.89 in this study (Figure 4D). Therefore, in this study, we chose the beta value (β = 7, Figure 4A) when we first approached the highest value to construct a gene network. WGCNA is shown to be very robust to β choice in terms of previously elucidated biological information. Then, adjacency was transformed to the topological overlap matrix (TOM) for measuring connectivity of network of the genes, which was defined to be sum of the adjacency of it and any other gene used for generation of networks (Yip and Horvath, 2007). In accordance with dissimilarity measure based on TOM within one 30 of smallest size (gene group) for genes dendrograms, a mean linkage hierarchical clustering was carried out to identify any gene that had the similar expression profile in gene modules.

### Identification of Important Clinical Modules

**FIGURE 5:**
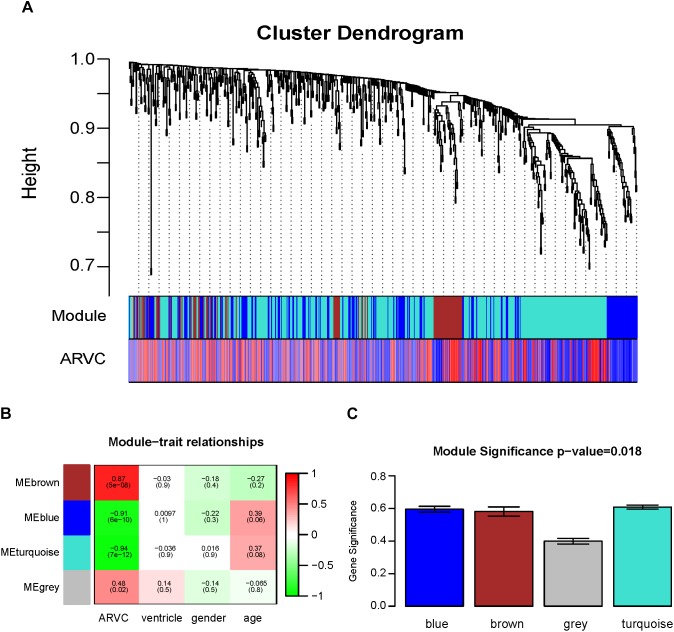
Identification of modules related to the clinical traits of the ARVC. **(A)** Dendrogram of all differentially expressed genes clustered on the basis of one dissimilarity measure (1-TOM). **(B)** Heatmap of the correlation between module eigengenes and ARVC clinical traits. **(C)** Distribution of the mean gene significance and errors in the modules related to ARVC state. Barplot of module significance (MS) defined as the average gene significance across all genes in modules.

Two methods were utilized for identifying ARVC state-related modules. Firstly, we defined gene significance (GS) to be log10 transformation of *P*-value within linear regression between ARVC state and gene expression. Additionally, module significance (MS) was defined to be mean GS for each gene within one module (Figure 5C). Generally, module that had absolute MS and ranked the first or second in the whole modules selected was regarded to be associated with clinical trait. Module eigengenes (MEs) were generally supposed to be the primary component within analysis of principal components for all gene modules, and each gene’s patterns of expression could be summarized in one singe characteristic expression profile in one given module. In the present study, we computed correlation between clinical trait and MEs for identifying related modules (Figure 5B).

### Differential Coexpression Analysis

The gene set net correlations analysis (GSNCA) (Rahmatallah et al., 2014) upon resultant modules was conducted for identifying the module which is differentially coexpressed between two ARVC sample and non-failing donor myocardial samples. The questions in this analysis test were gene sets’ identification expressed with various distributions, variances, means, or structure of correlation between two conditions. On the significance level of 0.05, pathways shows one statistical evidence that they are differentially coexpressed. GSNCA assigns one normalized eigenvector version of correlation matrix corresponding to biggest eigenvalue as one weight vector for genes within gene set under the two conditions. For ensuring appropriate indexing within modules by gene name within all significant pathways in this study, genes’ lists within each pathway are supposed to consist of only the available genes within the most relevant modules (brown and turquoise modules in WGCNA results). Any gene with not special mapping to the identifiers of gene symbol or does not exist within data set of the modules was abandoned from pathways. This ensures appropriate gene indexing within modules by gene name within all significant pathways. At last, we kept pathways only with 10 ≤ *n* ≤ 500 where *n* denotes the number of genes that remain within pathways after filtering procedures. Additionally, the GSNCA approach is part of Bioconductor GSAR package (Rahmatallah et al., 2017b).

The most highly correlated pathways in both ARVC and control samples, recognized utilizing structure of minimum spanning tree-2 (MST-2) carried out within package GSAR’s *plotMST2.pathway* function. One correlation network’s MST2 is formed through combination of the first and second MSTs as well as highlights minimum essential link set (i.e., highest correlation) between genes within coexpression network (Rahmatallah et al., 2014). The MST is defined to be acyclic tree that has shortest link which connects each gene within coexpression network. Within structure of MST2, genes with degree of highly associated are put into central position. The colors of nodes suggest weight factor (w)’s value assigned to all genes for reflecting the mean correlation with any other gene within gene set coexpression.

### Diagnostic Effectiveness Evaluation

Receiver operating characteristic (ROC) curves were set up for assessing areas under the curves (AUCs) that had 95% of CI. Results of AUC are regarded excellent for the values of AUC ranging between 0.9 and 1, good for the values of AUC ranging between 0.8 and 0.9 (El Khouli et al., 2009). Thus, when hub gene’s AUC value > 0.8, it was considered to have good specificity and sensitivity to distinguish between ARVC and healthy control group. We used this as one of the indicator for diagnostic effectiveness evaluation by maximizing Youden’s index, plotted ROC curve, and calculated the AUC with “ROCR” package (Sing et al., 2005). Then, comparative analysis on hub genes within two groups was analyzed by independent sample *t*-test at the significance level of 0.05.

## Results

### Identification of DEGs in ARVC Tissues

Figure 1 shows the research workflow. We obtained gene expression matrices from the training set GSE29819, including 6 ARVC specimens derived from heart transplantation candidates and 6 non-failing myocardial donor hearts myocardial samples after preprocessing of data and assessment of quality. Genes that have one stable expression across both groups were abandoned, as they provide little or no distinction. From ∼21,755 genes within dataset, a total of 619 DEGs (263 upregulated and 356 down-regulated) were selected for network construction under threshold of FDR < 0.05 and |log2FC|>1 (Supplementary Table S1). Figure 2B shows each gene’s volcano plot. A heatmap showing significantly DEGs can be found in Figure 3A. The score plots of PCA disclosed one tendency of intragroup aggregation and separation after unit variance scaling, normalization, and alignment of data (Figure 3B). The PCA score trajectory plots of ARVC did not substantially overlap with the profiles of the control group, indicating that the heatmap visualization and parallel PCA score trajectory plots both showed apparent differences resulting from the ARVC and control group.

### Weighted Coexpression Network Construction

Twelve ARVC samples that had clinical data were incorporated into coexpression analyses (Figure 2A). We used the “WGCNA” R package for putting DEGs that had similar patterns of expression to modules by the average linking clustering. In the present research, power of β = 7 (scale-free *R*^2^ = 0.89) was chosen to be soft-threshold (Figure 4).

Then, the hierarchical clustering tree for 619 DEGs was determined by conducting hierarchical clustering for dissTOM (Figure 5A), and we determined the most significant correlation modules with clinical features. Finally, a total of three modules related to the ARVC status were identified (Figure 5A and Supplementary Table S2), with the size of modules between 87 genes (brown modules) to 365 genes (turquoise modules). Additionally, 2 genes (*L3MBTL4* and *EPN2*) were assigned into the gray module (insignificant module), so they were omitted in subsequent analyses.

### Two Coexpression Modules Are Highly Correlated With ARVC State

Correlation between ARVC state and level of expression of the module eigengenes was calculated for identifying the most remarkable correlations. The highest positive correlation within module-feature relation was discovered between ARVC state and brown module (*r* = 0.87, *p* = 5e-08; Figure 5B), and the highest negative correlation between turquoise modules and ARVC state (*r* = -0.94, *p*_2_ = 7e-12; Figure 5B). Brown module includes 87 genes and the turquoise module includes 365 genes. MS between modules was also compared (Figure 5C), the result demonstrated that genes in brown or turquoise modules had highest positive or negative correlation with to ARVC status.

**FIGURE 6:**
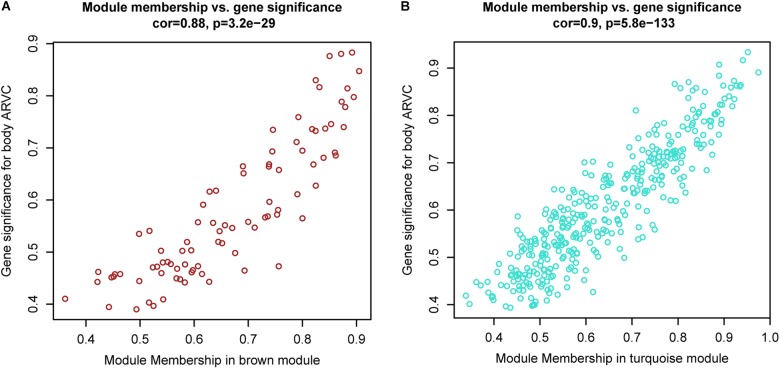
Scatterplots showing gene significance versus module membership for the brown **(A)** and turquoise **(B)** modules.

It was found that genes in modules were related to representative eigengene of it with various levels of module membership (MM), quantified by the module eigengene-based connectivity. For identifying the genes related to ARVC state, we defined measure of gene significance (GS) to be one Pearson gene expression correlation with ARVC state. The results showed one marked Pearson correlation between GS and MM values within brown module (correlation = 0.88, FDR corrected *p*-value = 3.2e-29, Figure 6A) as well as within turquoise module (correlation = 0.9, FDR corrected *p*-value = 5.8e-133, Figure 6B). The genes that were markedly related to ARVC state were usually the most important module members related to ARVC state. The input data of the WGCNA method in this study have been filtered for gene expression difference analysis using package Limma (Ritchie et al., 2015). The cut-off criteria are FDR < 0.05 and | log_2_^FC^| > 1. Therefore, the genes contained in the brown and turquoise modules satisfy the above conditions.

### Differential Coexpression Analysis Reveals Key Pathways and Hub Genes

**FIGURE 7:**
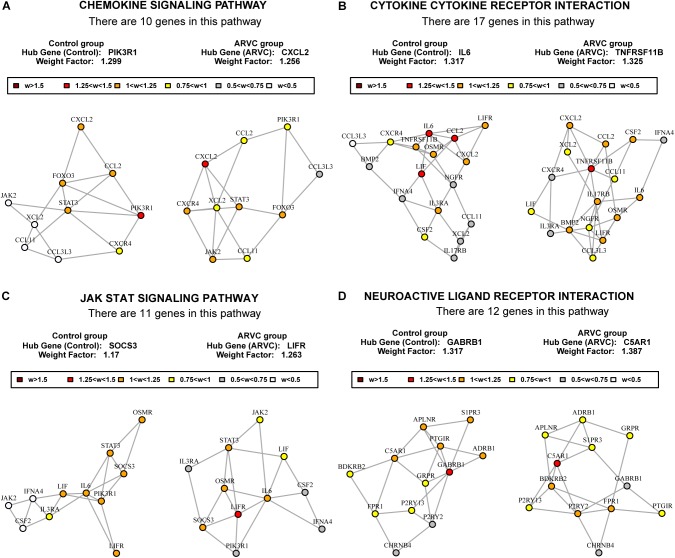
MST2s plot of the four key pathways correlation network. This plot was produced by package GSAR to illustrate the most highly correlated pathways and their hub genes of the two modules in both control samples and ARVC samples. Four ARVC state related biological pathways were identified by GSNCA **(A–D)**.

To find out the vital pathways and hub genes contained in the coexpression modules, we used the function GSNCA test (Rahmatallah et al., 2014) of GSAR package (Rahmatallah et al., 2017b). We found that GSNCA identified four key pathways and their hub genes (*p* < 0.05, Supplementary Table S3). The cytokine-cytokine receptor interaction, chemokine signaling pathway, JAK-STAT signaling pathway, and neuroactive ligand–receptor interaction were identified in the ARVC samples as key pathways (Figure 7). The MST2 plot for ARVC samples demonstrates that *CXCL2* is comparatively highly associated between with lots of other genes within the chemokine signaling pathway (Figure 7A). The same are true for the *TNFRSF11B, LIFR*, and *C5AR1* gene in the cytokine–cytokine receptor interaction pathway (Figure 7B), JAK-STAT signaling pathway (Figure 7C), as well as the neuroactive ligand–receptor interaction (Figure 7D), respectively. The *w*-values of them are decreased within control samples. The pattern may indicate regulatory roles of these genes in ARVC samples that are lost in the control samples. Even though the four genes lost the high *w*-values within control samples, these genes were still close to each other within structure of MST2 in control group, this indicates that correlation of them with other genes was not entirely lost, instead, it was reduced.

### Key Pathways and Hub Genes Identification and Validation

**FIGURE 8:**
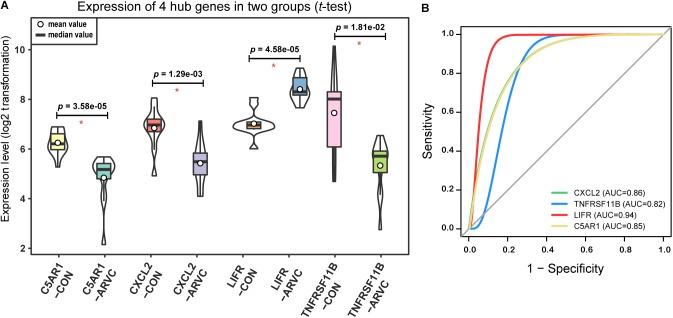
Hub genes validation. **(A)** Violin plot of the four correlated hub genes (*C5AR1, CXCL2, LIFR*, and *TNFRSF11B*) expression between ARVC group and control group. The violin plot showed the mean value, median value (while circle and black horizontal band, respectively), and range (black thin vertical line). The curve on the sides provided the approximate frequency distribution at each expression level. The *p*-values of the *t-*test were marked on the black lines. **(B)** ROC curves and AUC areas were used for evaluating the efficiency of diagnosis of these four hub genes (*C5AR1, CXCL2, LIFR*, and *TNFRSF11B*). They all be with high AUC areas (AUC > 0.8).

As we described earlier, after combined WGCNA and GSNCA test analysis filtering, the four key pathways and four genes (*CXCL2, TNFRSF11B, LIFR, and C5AR1*) in ARVC samples were identified, and they were entered *t*-tests subsequently, and their results showed significant differences (all *p* < 0.05, Figure 8A) between two groups. The AUC areas of the above genes were all greater than 0.8 (Figure 8B), and the four genes with a high correlation (|r| > 0.6, Table 1), suggesting that they might be good diagnostic biomarkers of ARVC.

**Table 1:** Potential hub genes related to the ARVC state.

Genes		Aliases	DEG analysis		Coexpression analysis	
			
			FC	FDR	p. weighted	cor. weighted
1	*CXCL2*	Macrophage Inflammatory Protein 2-Alpha	-2.84	2.24e-04	9.91e-05	-0.71
2	*TNFRSF11B*	Tumor Necrosis Factor Receptor Superfamily, Member 11b	-4.23	7.47e-04	6.14e-04	-0.65
3	*LIFR*	Leukemia Inhibitory Factor Receptor Alpha	2.80	4.00e-07	9.05e-09	0.89
4	*C5AR1*	Complement Component 5a Receptor 1	-2.79	8.63e-05	2.56e-05	-0.75


*The cor. weighted is the Pearson’s correlation of the Module Membership association. The highest correlations should be with the ARVC state (|*r*|= 0.6 to 0.8) based on previous study (Juniper et al., 2000).*

## Discussion

Sudden cardiac arrest might be ARVC’s first manifestation. It has a variable clinical course and is biologically heterogeneous. Therefore, understanding the molecular mechanisms of ARVC is crucial for early diagnosis, which helps make effective therapy of ICD. Endomyocardial biopsy is invasive, so obtaining myocardial tissue from ARVC patients is very difficult in clinical practice. In the research, we explored GSE29819 gene expression profile with 12 ARVC specimens (including all heart right and left ventricular myocardial samples) and 12 non-failing donor myocardial samples for exploring ARVC molecular mechanism and discover four hub genes and key pathways, which may be meaningful candidate therapeutic targets based on bioinformatic analysis.

As far as we know, this is the first attempt to combine WGCNA with GSNCA methods for identifying hub genes to be biomarkers able to distinguish ARVC from the non-failing control group. In this study, the results showed that there were a total of 619 DEGs (263 upregulated genes and 356 downregulated genes) between non-failing donor myocardial samples and ARVC tissues (FDR < 0.05). The PCA score trajectory plots showed apparent differences resulting from ARVC and the control group. WGCNA analysis used coexpression patterns to identify the two gene modules (brown and turquoise) as being most significantly associated with ARVC state. The sample traits obtained from our raw data were ARVC status, gender and ventricle, follow by the gene modules (brown and turquoise) related to ARVC status were further analyzed according to the prompts in Figure 5. Then the ARVC-related four key biological pathways (cytokine–cytokine receptor interaction, chemokine signaling pathway, JAK-STAT signaling pathway, and neuroactive ligand–receptor interaction) and four hub genes (*CXCL2, TNFRSF11B, LIFR*, and *C5AR1*) in ARVC samples were identified by GSNCA method. Together, the results suggest that the new candidate genes from modules might be added on biomarker list of ARVC state that is known at present, and hence suggest that the genes and their pathways require further analysis.

This study used two bioinformatics analysis methods, WGCNA and GSNCA. WGCNA is a system biology method that constructs a coexpression network on the basis of expression profiles’ similarity within a sample, providing global interpretation of gene expression information (Langfelder and Horvath, 2008). The WGCNA algorithm was utilized for identifying the involved biological pathways, disease-related genes, and therapeutic targets, like a familial combination of hyperlipidemia (Plaisier et al., 2009), pemphigus and systemic lupus erythematosus (Sezin et al., 2017), and rheumatoid arthritis (Sumitomo et al., 2018). Briefly, WGCNA is designed to uncover highly correlated gene modules and to relate gene clusters to one another and to sample traits. In recent studies, it has been verified in previous sample characteristics such as disease status (Yan et al., 2018), gender (Fatima et al., 2018), age (Maffei et al., 2017), and BMI (Wang W. et al., 2017). It was also proved that it was a reliable and promising instrument for cardiovascular diseases’ clinical diagnosis (Chen et al., 2016) and cardiomyocyte differentiation (Liu et al., 2017). The second method is GSNCA. Currently, although GSA methods have been published in some literature, their test hypotheses have not been fully studied. For instance, Gene Set Coexpression Analysis (GSCA) aggregates differences of pairwise correlations between the two conditions coexpression (Choi and Kendziorski, 2009), while other approaches, like differentially coexpressed gene sets (dCoxS), aggregate differences within relative entropy (Cho et al., 2009). In this study, package GSAR (Rahmatallah et al., 2017b) offers one set of approaches to testing multivariate null hypothesis against particular alternatives, which including the net correlation structure (function GSNCA test). GSNCA test is a method for analysis on differentially coexpressed pathways, and evaluates significance of genes within pathways as well. It examines the concordance and regulatory relations between expressions of genes vary between the phenotypes based on unchanged net correlation structure (Rahmatallah et al., 2014). Other approaches, like Coexpression Graph Analysis (CoGA) identify co-expressed gene sets through testing spectral distribution equality, it compares two networks’ structural property by using Jensen-Shannon divergence to be one measure of distance between the distribution of graphs, and establishes one full network from the pairwise correlation coexpression (Santos Sde et al., 2015; de Assis et al., 2018). However, in GSNCA, differences do not exist within vectors of gene weight between the two conditions, and MSTs of correlation network are used for examining changes of correlation structure of one gene set between the two conditions, and the most influential (hub) genes are highlighted (Rahmatallah et al., 2014). This is the methodological basis used in this study (Figure 7) and previous researches (Mousavian et al., 2017; Rahmatallah et al., 2017a).

The balance between apoptotic and protective mechanism of cardiomyocytes can be decided by some signaling pathway networks. According to the GSNCA analysis on the genes within two modules (Figure 7), we found that the ARVC state related biological pathways were significantly enriched in the cytokine–cytokine receptor interaction, chemokine signaling pathway, JAK-STAT signaling pathway, and neuroactive ligand–receptor interaction pathway. An amount of research mentioned the close relation between the above processes and cardiomyopathy or cardiovascular disease. At present, the first three pathways have been confirmed in other non-ARVC cardiomyopathy. Chemokine receptors and chemokines control leukocyte migration in process of inflammation and they are involved into heart inflammation and dysfunction, and cardiac myocytes themselves also can produce inflammatory mediators (Nian et al., 2004; Rohini et al., 2010). Some studies reported that the genetic variants of chemokine receptors and chemokines are weakly but importantly related to chagasic cardiomyopathy development (Cunha-Neto et al., 2009; Florez et al., 2012), an inflammatory dilated cardiomyopathy that is. Additionally, Pilichou et al. (2009) demonstrated that the necrosis of myocytes is principal initiator of the myocardial damage within ARVC, which includes one inflammatory response as well as massive calcification in myocardium, followed by repair of injury that has replacement of fibrous tissue, and the myocardial atrophy. Asimaki et al. (2011) also reported that some cytokines from the myocardium might be involved in the destruction of desmosomal proteins and arrhythmias in ARVC. These studies demonstrate the relevance of chemokines and cytokine receptors in cardiomyopathy, and increase the reliability of this study with regard to the frequent manifestations of differential enrichment in chemokine and inflammatory factor pathways. In addition, previous study has shown that alteration within Janus kinase (JAK)-signal transducer and activator of transcription (STAT) signaling within patients suffering from end stage dilated cardiomyopathy (Podewski et al., 2003). JAK/STAT pathway can effect cardioprotective or proapoptotic gene expression (Gross et al., 2006). Recent studies have shown that cardiac fibrosis and heart failure can be attenuated by JAK/STAT signaling pathway (Al-Rasheed et al., 2016; Liu et al., 2018). The above studies link the JAK/STAT pathway to the cardiomyopathy. Therefore, early attention to the balance of relevant pathways can improve long-run prognosis of people suffering from cardiomyopathy, including those with ARVC. Although the correlation between the last neuroactive ligand receptor interaction pathway and cardiovascular disease has not been reported, this pathway may be a new idea for further study of the ARVC mechanism.

In addition, the four hub genes (*CXCL2, TNFRSF11B, LIFR*, and *C5AR1*) with a high correlations (|r| > 0.6, Table 1) were associated with these above four vital signaling pathways in ARVC samples (Figure 7). CXCL2 (also known as macrophage inflammatory protein, MIP-2) is part of one chemokine superfamily encoding secreted proteins that are involved into inflammatory and immunoregulatory process, and released by various cells to respond toward injury or infection, and was detected originally within the macrophages to one part of the responses toward the inflammatory stimuli (Charo and Ransohoff, 2006). Recent genome-wide association studies have recognized CXCL2-related loci related to coronary artery disease risk (McPherson and Davies, 2012). Its role in the cardiovascular field is mostly related to inflammation, it suggested an abnormality in the inflammatory state of ARVC patients, which was also consistently with the reported that patchy inflammatory infiltrates in the right ventricular using autopsy and myocardial biopsy (Campuzano et al., 2012). *TNFRSF11B* is member 11B, tumor necrosis factor receptor superfamily, expressed within lymphoid cells as well as up-regulated by the stimulation of CD40, involved within the osteoclastogenesis, it is recognized as one candidate cardiovascular disease gene by human protein atlas, and it might also work in arterial calcification prevention (Harper et al., 2016). C5AR1 (Complement C5a Receptor 1) is an important member of the complement system. Studies reported that modulation of complement system could serve as one target for arrhythmogenic cardiomyopathy treatment (Mavroidis et al., 2015), and its signaling pathway upon blood macrophages/monocytes plays one role of pathology in Ang II-induced cardiac remodeling and inflammation (Zhang et al., 2014). *LIFR* gene encodes one protein belonging to the family of type I cytokine receptors. Previous studies have reported that *LIFR* gene is one of the important differential genes in the JAK-STAT signaling pathway in people suffering from end stage dilated cardiomyopathy (Podewski et al., 2003). LIF and its receptor also involved in modulating endoderm of embryoid bodies to promote cardiomyogenesis (Bader et al., 2001). Therefore, we suspect that the performance of myocardial cell electrophysiological disorders and cardiac remodeling deficiency in ARVC patients may be related to the above genes. Although the role in ARVC studies of the above hub genes had not yet been demonstrated, their necessary functions and related pathways in the cardiovascular field was emphasized in the results of this study and provided further insights into exploration in the ARVC field. However, further experimental work is required for establishing which candidate discussed above predominantly contributes to ARVC state.

Similarly, our study also has some limitations. Firstly, our conclusions are capable of being applied into one restricted population in ARVC patients as well as non-failing heart donors, but it is not proper to apply our findings to other non-ARVC patients with similar symptoms, including dilated cardiomyopathy patients. Secondly, histopathological changes in ARVC are progressive procedure. The participant samples with ARVC in this research were prepared from the hearts explanted in orthotopic heart transplantation representing the myocardium from patients with end stage heart failure. Our research lacked early-stage of ARVC patients; thus, the results may not be representative with they are applied into ARVC differential diagnosis at early stages. At last, only one genetic dataset from human ARVC heart tissues, the GSE29818 dataset, was searched from the web databases and analyzed in our study, we lacked rigorous testing data. Despite this, the hub gene analysis also gave further research directions for the diagnosis of ARVC.

## Conclusion

In conclusion, our research described hub genes as well as key pathways which might be involved into ARVC diagnosis, utilizing bioinformatics-based WGCNA combine with GSNCA for constructing gene coexpression networks. Even though further experimental validation is required for the implication by association, our findings demonstrate that the computational methods based on systems biology might complement traditional gene-wise approaches, and as such, might offer a new insight in therapeutic intervention within rare diseases of people like ARVC.

## Author Contributions

PC contributed in conceptualization, methodology, data curation, writing, original draft preparation, and visualization. PC and YX contributed in formal analysis. PC and WW contributed in writing the review and editing. BL, WW, and SZ contributed in supervision. WW and SZ contributed in project administration. WW and YZ acquired the funding.

## Conflict of Interest Statement

The authors declare that the research was conducted in the absence of any commercial or financial relationships that could be construed as a potential conflict of interest.

## Footnotes

1 http://www.ncbi.nlm.nih.gov/geo/

## Supplementary Material

TABLE S1 619 DEGs in ARVC tissues.

TABLE S2 DEGs in WGCNA modules.

TABLE S3 Result_MST of GSNCA.
